# Kinematic and muscle synergy patterns of the lower limbs during jump-landing with side-cutting in individuals with functional ankle instability

**DOI:** 10.3389/fbioe.2025.1739185

**Published:** 2026-01-13

**Authors:** Xinqi Ji, Xiaoliang Li, Lijing Yu, Yongyue Song

**Affiliations:** 1 School of Physical Education, Hebei Normal University for Nationalities, Chengde, China; 2 School of Sport Training, Tianjin University of Sport, Tianjin, China; 3 Department of Physical Education, Chengde Medical University, Chengde, China

**Keywords:** coper, functional ankle instability, jump-landing side-cutting, muscle synergy, neuromuscular control

## Abstract

**Objective:**

This study investigated lower-limb kinematics and neuromuscular control in individuals with functional ankle instability (FAI) during a standing long jump-landing side-cut tasks, and compared them with Copers (individuals with ankle sprain history but no persistent instability) and healthy controls to reveal synergy reorganization mechanisms underlying FAI and inform rehabilitation strategies.

**Methods:**

Ten participants were included in each group (FAI, Coper, and control). For the jump-landing side-cut task, participants stood 80 cm behind a force plate, jumped forward maximally with both legs, landed on one test leg at the plate center, then immediately side-cut 30° to the opposite side of the test leg (lateral distance ≥80 cm from the plate). Lower-limb kinematics and electromyography were recorded during the task using a synchronized motion capture and EMG system. Muscle synergies were extracted via non-negative matrix factorization (NNMF, 90% variance accounted for as termination criterion) to compare synergy number, activation timing, and muscle contributions among groups.

**Results:**

(1) The FAI group exhibited significantly greater knee and ankle flexion-extension and hip abduction angles compared with the Coper and control groups, while the Coper group showed a larger ankle range of motion than controls (p < 0.05). (2) All three groups demonstrated four common synergy modules. (3) The early synergy (Module 1) activation duration was shorter in the FAI and Coper groups than in controls, whereas the late synergy (Module 4) lasted longer in the FAI group (p < 0.05). (4) Significant differences in muscle weightings were observed among groups across modules (p < 0.05).

**Conclusion:**

Individuals with FAI adopt a protective movement strategy characterized by increased flexion and abduction to enhance stability. Muscle synergy analysis reveals an asymmetric activation pattern with reduced early activation, prolonged late compensation, and a proximal-dominant, delayed distal control pattern. Although the Coper group demonstrates movement characteristics more similar to healthy controls, mild over-flexion and delayed responses remain.

## Introduction

The ankle joint is one of the most important load-bearing and supporting joints of the human body, playing a crucial role in maintaining movement stability, coordination, and the transmission of lower limb dynamics (Gomes et al., 2023). With the growing popularity of sports participation and the rising level of athletic performance, ankle injuries—particularly lateral ligament sprains—have become one of the most common types of acute musculoskeletal injuries (Dai et al., 2019). Statistics show that in multidirectional sports such as track and field, basketball, and soccer, ankle sprains rank first among all sports-related injuries (Waterman et al., 2010). Approximately 40% of individuals experience persistent instability, pain, or recurrent sprains following the initial injury, leading to the development of Chronic Ankle Instability (CAI) (Yeung et al., 1994). Long-term CAI impairs daily and sports function, triggers joint degeneration and kinetic chain issues, and poses ongoing challenges to performance and rehabilitation. (Arnold et al., 2011).

CAI generally consists of two primary subtypes: Mechanical Ankle Instability (MAI) and Functional Ankle Instability (FAI) (Kobayashi and Gamada, 2014). FAI is characterized by recurrent “giving way,” a subjective sense of instability, and deficits in neuromuscular coordination—despite the absence of obvious mechanical damage (Miklovic et al., 2018). FAI is often accompanied by impaired neural control and decreased proprioceptive function, and abnormal motor pattern adaptations (Wang et al., 2021). Evidence indicates that FAI arises not only from initial lateral ligament injury but also from complex neuromuscular regulation deficits, including deterioration in peripheral receptor function, delayed spinal reflex pathways, and cortical–cerebellar reorganization (Kim and Choi, 2016). These neurological and functional impairments make FAI a distinct clinical entity that differs from MAI in pathogenesis and rehabilitation needs. Given these dual functional and neurological characteristics, traditional rehabilitation methods focusing primarily on mechanical stabilization or strength training often fail to achieve complete recovery (Y, 2015; De Fazio et al., 2025). Therefore, exploring the neural control mechanisms underlying FAI has significant theoretical and clinical value.

In recent years, research on FAI has progressively shifted from a joint-based perspective toward neural mechanisms. Numerous studies have shown that individuals with FAI exhibit altered kinematic and electromyographic patterns during dynamic tasks such as postural control, gait, and jumping (Jeon et al., 2021; Zhong et al., 2025; Zhao et al., 2025). For instance, during single-leg landing tasks, FAI individuals commonly display limited ankle dorsiflexion range, reduced eversion response, increased knee flexion angles, and decreased peak ground reaction forces—reflecting a potential movement adjustment to maintain balance under perturbations (Kim et al., 2019; Beltrame et al., 2023). However, such compensatory patterns often reduce movement efficiency and may further impair the ankle’s ability to respond rapidly to external disturbances. Neurophysiological evidence further demonstrates delayed peroneal muscle activation and prolonged muscle latency in these individuals, suggesting weakened reflexive responsiveness (Hoch and Mckeon, 2014). Consequently, FAI is widely recognized as a systemic functional disorder stemming from neural control deficits.

Under complex dynamic conditions—such as standing long jump-landing and side-cutting movements—the ankle experiences substantial vertical and lateral forces (up to 3–5 times body weight during landing), and rapid multidirectional displacements, placing greater demands on the temporal and spatial coordination of the neuromuscular control system (Simpson et al., 2020). Compared with static balance tests, these tasks simulate real-world sports scenarios and provide a more ecologically valid evaluation of dynamic control capacities and injury-prevention strategies (Doherty et al., 2016). For individuals with FAI, the combined challenges of landing impact absorption and sudden direction change during side-cutting exacerbate the deficits in proprioceptive feedback and neuromuscular coordination, making this task a sensitive indicator of functional instability (Wright et al., 2016). Therefore, examining standing long jump-landing side-cut tasks allows for a comprehensive assessment of FAI individuals’ kinematic alterations and their central nervous system’s timing and coordination adaptations under multidirectional loading. In this study, a three-dimensional motion capture system and surface electromyography were employed to analyze the kinematic and neuromuscular characteristics of individuals with FAI during these tasks, providing insights into proprioceptive deficits and neural coordination adaptations.

Traditional electromyographic (EMG) analyses often focus on single muscles, limiting their capacity to reveal the global coordination among multiple muscle groups (Beltrame et al., 2023). Recently, Muscle Synergy Analysis based on Non-negative Matrix Factorization (NNMF) has become widely used in motor control and rehabilitation research. This approach posits that complex motor behaviors can be represented by a limited number of synergy modules, each reflecting a group of muscles that are co-activated in a specific temporal pattern (Lee and Seung, 1999). Such modular control reflects the central nervous system’s strategy for simplifying control dimensions to achieve efficient movement execution (Matsuura et al., 2024). Investigating the muscle synergy patterns of FAI individuals can elucidate whether their neural control system undergoes structural or temporal reorganization, and whether such changes are maladaptive or compensatory. Previous findings suggest that while the number of synergies does not necessarily decrease in FAI, their activation timing becomes desynchronized, with an increased contribution from proximal muscles—indicating a possible “temporal compensation” mechanism within the Central Nervous System (CNS) in response to reduced peripheral sensory input (Kim et al., 2023).

Approximately 60% of individuals who experience an initial ankle sprain do not develop recurrent injury or instability; these individuals are referred to as Copers (Wikstrom and Brown, 2014). Though Copers have sustained ligament injuries, they are able to regain functional stability within a short period and perform similarly to healthy individuals in clinical and biomechanical assessments. They represent a transitional group between healthy and FAI individuals, reflecting neuromuscular adaptation and reorganization following injury. Comparative studies between Copers and FAI subjects can thus reveal distinct neural plasticity pathways and stages of motor control recovery, providing valuable implications for rehabilitation strategies.

Collectively, the current evidence indicates that the pathogenesis of FAI involves multiple interacting factors, including proprioceptive deficits, altered muscle activation, kinematic adaptations, and central neural reorganization. Analyses limited to one dimension cannot fully capture this complexity. Particularly in dynamic tasks such as landing and cutting, the interplay between joint kinematics and temporal coordination of muscle synergies is essential for maintaining stability. Therefore, this study integrates kinematic and muscle synergy analyses to examine differences among FAI, Coper, and healthy individuals during standing long jump-landing side-cut tasks. The objectives are to: (1)determine how FAI individuals restructure lower-limb kinematic patterns during landing and side-cutting; (2) explore whether their neural control systems exhibit spatiotemporal reorganization reflected by altered muscle synergy composition, activation timing, and modular coordination, as well as associated kinematic adjustments; and (3) identify whether Copers achieve functional recovery through specific neuromuscular adaptations.

## Methods

### Participants

A total of 30 participants were recruited, with 10 assigned to the Functional Ankle Instability (FAI) group,10 to the Coper group (individuals with a history of ankle sprain but no current instability symptoms), and 10 to the healthy control group (CON). To eliminate the potential effects of sex and limb dominance, only male participants with the dominant limb corresponding to the affected side were included. The demographic characteristics of the participants are summarized in Table1.

**TABLE 1:** Basic Information of the Subjects.

	FAI	Coper	CON
Number of participants	10	10	10
Age (years)	21.5 ± 2.4	21.7 ± 2.3	21.3 ± 2.4
Height (cm)	173.4 ± 3.0	175.2 ± 3.5	174.3 ± 3.3
Body weight (kg)	69.7 ± 6.7	71.2 ± 3.7	72.3 ± 5.4
CAIT score (points)	16.5 ± 2.5	25.4 ± 1.0	29.4 ± 1.3
IdFAI score (points)	21.5 ± 3.2	0	0

FAI, functional ankle instability group; Coper, Coper group; CON, healthy control group; CAIT, cumberland ankle instability tool; IdFAI, identification of functional ankle instability.

### Inclusion criteria

FAI were required to meet the criteria for CAI while excluding MAI (Gribble et al., 2013). The inclusion criteria for the FAI group were as follows:

1. A history of at least one severe lateral ankle sprain accompanied by pain and swelling, requiring immobilization and/or non-weight bearing for at least 3 days.
2. The ankle sprain occurred more than 1 year prior to participation.
3. Repeated episodes of ankle sprain and/or feelings of instability occurring at least twice within the past 6 months or recurrent instability within 3 months prior to testing.
4. Cumberland Ankle Instability Tool (CAIT) score<24 and Identification of Functional Ankle Instability (IdFAI) score>11 on the injured side (Wang et al., 2021; Li et al., 2019).
5. Negative results in the anterior drawer and talar tilt tests.

Participants in the Coper group met the following criteria:

1. A history of at least one severe lateral ankle sprain requiring immobilization and/or restricted weight bearing for at least 3 days.
2. The injury occurred more than 1 year prior to testing.
3. No pain, recurrent sprains, instability, or weakness in the previously injured ankle during the past year.
4. CAIT≥24 and IdFAI≤10.
5. A full return to pre-injury activity level for at least 12 months.

Participants in the CON group met the following criteria:

1. No history of ankle sprain or instability.
2. CAIT≥24 and IdFAI≤10.

All participants reported more than 90 min of regular physical activity per week. Both FAI and Coper participants had sustained ankle sprains only on their dominant limbs.

### Exclusion criteria

Exclusion criteria included:

1. History of musculoskeletal surgery involving the lower limbs (bones, joints, or nerves).
2. History of fracture in the lower extremities.
3. Any acute lower-limb musculoskeletal or joint injury within the past 3 months.
4. Presence of visual or vestibular dysfunction.
5. Positive results in the anterior drawer or talar tilt tests.
6. Participation in any other interventional clinical study within the past 3 months.

To minimize the effects of fatigue, all participants were instructed to avoid strenuous physical activity within 24 h prior to testing.

The study was approved by the Ethics Committee of Capital University of Physical Education and Sports (Approval No.2024A121). All participants read and signed written informed consent before taking part in the experiment.

### Equipment and data acquisition

A three-dimensional motion capture system (OptiTrack, 8-camera, NaturalPoint, United States) was used to record the spatial coordinates of anatomical landmarks at a sampling rate of 200 Hz. Marker placement followed the system’s Conventional Full-Body 39-marker model.

Ground reaction force (GRF) data were collected using a force platform (Kistler 9260AA, Switzerland) at a sampling frequency of 1,000 Hz.

EMG signals were acquired using a wireless EMG system (Delsys Trigno, United States) at a sampling rate of 2000 Hz. A total of 11 sensors were placed on the dominant lower limb of each participant over the following muscles: rectus femoris (RF), vastus lateralis (VL), vastus medialis (VM), peroneus longus (PL), tibialis anterior (TA), gluteus maximus (GMAX), biceps femoris (BF), semitendinosus (ST), medial gastrocnemius (GM), lateral gastrocnemius (GL), and soleus (SL).

The motion capture system, force plate, and EMG system were synchronized during all testing procedures to ensure temporal alignment and accurate integration of kinematic, kinetic, and electromyographic data.

### Experimental procedures and task description

Upon arrival at the laboratory, participants were informed of the experimental procedures and signed written informed consent. Each participant performed a warm-up session on a treadmill for no less than 5 min, followed by at least three practice trials of the test movement to ensure familiarity with the task. The examiner cleaned the skin surface of target muscles with alcohol wipes to remove sweat and facilitate optimal EMG signal quality. EMG sensors were then attached, after which participants changed into motion capture suits and had reflective markers affixed according to the full-body model.

**FIGURE 1:**
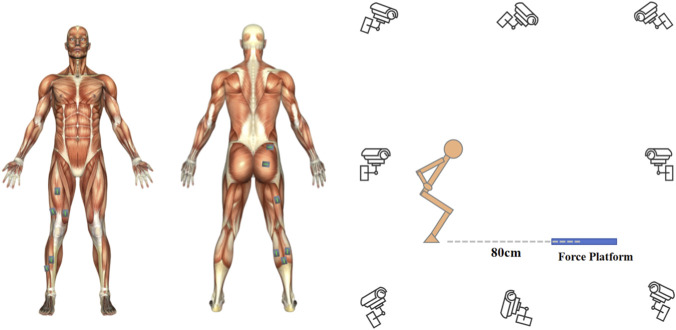
Experimental setup and EMG electrode placement. The dominant lower limb was instrumented with 11 surface EMG electrodes placed over the following muscles: RF (rectus femoris), VL (vastus lateralis), VM (vastus medialis), PL (peroneus longus), TA (tibialis anterior), GMAX (gluteus maximus), BF (biceps femoris), ST (semitendinosus), GM (medial gastrocnemius), GL (lateral gastrocnemius), and SL (soleus).

The experimental task involved a jump-landing side-cutting maneuver (Son et al., 2017). Participants stood with both feet approximately 80 cm behind the center of the force plate. They were instructed to jump forward and upward maximally using both legs, landing only on the test leg at the center of the force plate, and immediately performing a side-cut movement toward the contralateral direction of the test leg (the cutting angle was approximately 30°). The lateral cutting distance after landing was required to be no less than 80 cm from the plate center. Each participant performed the task until 6 successful trials were recorded, with a minimum 30-s rest interval between successive trials to minimize fatigue (Figure 1).

### Data processing

#### Data segmentation

The vertical ground reaction force (vGRF) was used to identify the start and end of each trial. The first frame in which the vGRF exceeded 10N was designated as the landing onset, marking the start of the movement. The last frame in which the vGRF dropped below 10N was defined as the take-off frame, marking the end of the movement. The segmented motion capture and force plate data were then processed using a 6 Hz low-pass Butterworth filter before further analysis.

#### Kinematic data processing

Kinematic analysis was conducted using OpenSim 4.4, and the workflow comprised three main steps:

1. Data Conversion: Raw motion capture data in. c3d format were converted into OpenSim-compatible. mot files using the C3D File Converter plug-in.
2. Model Scaling: A Full-Body full-body lumbar spine model (FBLS) was scaled to match each participant’s anthropometric characteristics (height, body mass, and segment lengths derived from marker positions). The Scale Model tool was used with the following criteria: total marker error<4 cm and root mean square (RMS) error <2 cm, ensuring high anatomical fidelity of individualized models.
3. Inverse Kinematics (IK) Analysis: The Inverse Kinematics tool was applied to compute joint angles of the hip, knee, and ankle. In this study, the angular definitions of the hip, knee, and ankle joints adhere to anatomical standards and OpenSim 4.4 specifications, with the anatomical neutral position as 0°: in the sagittal plane (flexion-extension), the hip and knee joints use flexion as positive and extension as negative, while the ankle joint uses dorsiflexion (flexion) as positive and plantarflexion (extension) as negative; in the frontal plane, the hip joint uses abduction as positive and adduction as negative, the knee joint uses valgus as positive and varus as negative, and the ankle joint uses eversion as positive and inversion as negative.

#### EMG data processing

All EMG preprocessing was performed in MATLABR2022a. The raw EMG signals were first band-pass filtered using a fourth-order Butterworth filter (10–400 Hz) to remove motion artifacts and high-frequency noise. The filtered signals were then full-wave rectified and subsequently low-pass filtered at 10 Hz (fourth-order Butterworth) to obtain the linear envelope. The EMG envelope was computed using a 0.125 s moving window with no overlap to smooth the signal before normalization. Each EMG channel was amplitude-normalized to its maximum envelope value across all trials to enable inter-muscle and inter-subject comparison.

All kinematic, kinetic, and EMG data were time-normalized to 101 points, representing the 0%–100% of the movement cycle, to allow for consistent temporal comparison across subjects and conditions.

### Muscle synergy analysis

The classical Gaussian NNMF algorithm extracted muscle synergies. In this method, the muscle activation model represents the sEMG signals, which can be decomposed into two matrices. One of them is the muscle synergy vector matrix (W, relative weights of muscles within each module) and the other is the time-varying activation coefficient (C). The decomposition model can be expressed as the following formula:

$$V_{\text{mn}}\approx {\left(\text{WC}\right)}_{\text{mn}}=\sum_{i=1}^{k}W_{\text{mi}}C_{\text{in}}=V_{\text{mn}}^{\prime }$$ (1)

In Formula 1, $V_{\text{mn}}$ represents m channels EMG signals with n sampling points, k represents the number of synergy modules and W reflects the activation weight of each muscle in the *i*th synergy modules. In this study, the W value is normalized to 0–1. When the weight values of these muscles are above 0.3, they are considered as main synergistic muscles (MSMs). C is the time-varying activation coefficient, which represents the contribution of the *i*th synergy matrices to the movement at time t. The portion where the C exceeded 0.3 was defined as the active phase of muscle synergy activation. $V_{\text{mn}}^{\prime }$ represents the reconstructed EMG signals.

The Variance Accounted For (VAF) was utilized to identify the optimal number of synergy modules k (Kattla and Lowery, 2010). The VAF can be expressed as:

$$\text{VAF}=1‐\frac{\text{RSS}}{\text{TSS}}=1‐\frac{\sum {\left(V_{\text{mn}}‐V_{\text{mn}}^{\prime }\;\right)}^{2}}{\sum {\left(V_{\text{mn}}\right)}^{2}}$$ (2)

In Formula 2, RSS is the residual sum of squares and TSS is the total sum of squares. The number of synergies was selected so that the VAF was at least 90% (Wang et al., 2025). To identify the characteristic muscle synergy patterns within each group, k-means clustering analysis was performed on all synergy modules obtained from the participants in each group. The optimal number of clusters was determined based on the elbow point of the scree plot, which represents the point where further increasing the number of clusters yields minimal reduction in within-cluster variance. If multiple synergy modules from the same participant were assigned to the same cluster, Pearson’s correlation coefficients were calculated between each module and the mean synergy pattern of that cluster (Munoz-Martel et al., 2021). Among these, the module with the highest positive correlation coefficient was selected for subsequent analysis to ensure representative consistency within each cluster.

NNMF analysis was performed in MATLAB R2022a. For each synergy number, the NNMF algorithm was randomly initialized 10 times with initial matrix values between 0.01 and 1, and the solution with the highest *R* ^2^ value was selected. The iteration terminated when the change in *R* ^2^ over 20 iterations was less than 0.01% of the current *R* ^2^, or when the maximum iteration number of 1,000 was reached.

### Analysis parameters

The analysis included both kinematic and muscle synergy indicators. Kinematic indicators consisted of joint angles of the hip, knee, and ankle in the sagittal plane (flexion–extension) and frontal plane (adduction–abduction). Muscle synergy indicators included the number of synergy modules extracted, the peak timing of each module’s C, the duration of activation for the C, and the MSM within each synergy module.

### Statistical analysis

Statistical analyses were performed using SPSS version 26.0 (IBM Corp., Armonk, NY, United States). The Shapiro–Wilk test was used to assess the normality of each variable, and all data were confirmed to follow a normal distribution. All results are expressed as mean ± standard deviation (Mean ± SD). One-way analysis of variance (ANOVA) was conducted for each parameter. Considering that a total of 49 ANOVA tests were performed, the family-wise error rate was controlled using the Bonferroni correction, resulting in an adjusted overall significance level of p = 0.05/49 = 0.001. Accordingly, differences were considered statistically significant at p < 0.001. Bonferroni *post hoc* tests were further applied for pairwise comparisons between groups, with the significance level set at p < 0.05 for multiple comparisons.

## Results

### Kinematic analysis

#### Sagittal plane joint angles

**FIGURE 2:**
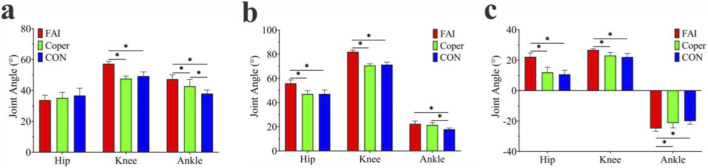
Sagittal plane joint angles. **(a–c)** Represent joint range of motion, maximum joint angle, and minimum joint angle, respectively. FAI, Functional Ankle Instability group; Coper, Coper group; CON, healthy control group; * indicates that the p-value from multiple comparisons <0.05.

The joint ranges of motion (ROM) are presented in Figure 2a. For the hip joint (FAI: 33.74° ± 3.24°, Coper: 35.12° ± 3.64°, CON: 36.72° ± 4.74°), no significant difference was found among the three groups (F = 1.443, p > 0.001, η^2^ = 0.097). For the knee joint (FAI: 57.23° ± 1.64°, Coper: 47.66° ± 1.62°, CON: 49.27° ± 2.77°), a significant difference was observed (F = 60.825, p < 0.001, η^2^ = 0.818), with the FAI group showing greater ROM than the Coper and CON groups (p < 0.05). For the ankle joint (FAI: 47.27° ± 2.86°, Coper: 42.77° ± 4.45°, CON: 37.93° ± 2.40°), a significant difference was also found (F = 19.413, p < 0.001, η^2^ = 0.590); the FAI group had greater ROM than both the Coper and CON groups, and the Coper group was greater than the CON group (p < 0.05).

The maximum joint angles are shown in Figure 2b. For the hip joint (FAI: 55.87° ± 2.89°, Coper: 47.12° ± 2.85°, CON: 47.41° ± 3.36°), a significant difference was found among the three groups (F = 26.659, p < 0.001, η^2^ = 0.664), with the FAI group significantly greater than the Coper and CON groups (p < 0.05). For the knee joint FAI: 81.89° ± 1.55°, Coper: 70.69° ± 1.45°, CON: 71.30° ± 2.16°), a significant difference was observed (F = 129.36, p < 0.001, η^2^ = 0.906), with the FAI group greater than both the Coper and CON groups (p < 0.05). For the ankle joint (FAI:22.41° ± 2.44°, Coper:21.48° ± 2.12°, CON:17.91° ± 1.11°), a significant difference was also found (F = 14.446, p < 0.001, η^2^ = 0.517), with the FAI and Coper groups greater than the CON group (p < 0.05).

The minimum joint angles are shown in Figure 2c. For the hip joint (FAI: 22.12° ± 2.60°, Coper: 11.99° ± 3.42°, CON: 10.69° ± 2.69°), a significant difference was found (F = 45.633, p < 0.001, η^2^ = 0.772), with the FAI group greater than the Coper and CON groups (p < 0.05). For the knee joint (FAI: 26.66° ± 0.86°, Coper: 23.03° ± 2.03°, CON: 22.03° ± 2.44°), a significant difference was observed (F = 16.374, p < 0.001, η^2^ = 0.565), with the FAI group greater than the Coper and CON groups (p < 0.05). For the ankle joint (FAI:−24.86° ± 1.87°, Coper:−21.28° ± 3.25°, CON:−20.01° ± 2.07°), a significant difference was also found (F = 10.337, p < 0.001, η^2^ = 0.434), with the FAI group smaller than the Coper and CON groups (p < 0.05).

#### Frontal plane joint angles

**FIGURE 3:**
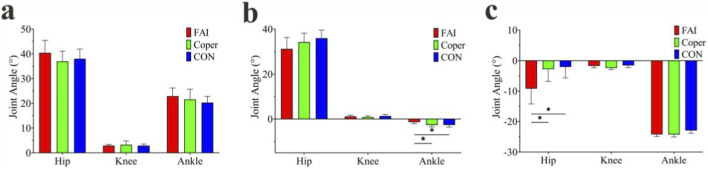
Frontal plane joint angles. **(a–c)** Represent joint range of motion, maximum joint angle, and minimum joint angle, respectively. FAI, Functional Ankle Instability group; Coper, Coper group; CON, healthy control group; * indicates that the p-value from multiple comparisons <0.05.

The joint ROM are presented in Figure 3a. For the hip joint (FAI: 40.42° ± 5.01°, Coper: 37.01° ± 4.14°, CON: 38.04° ± 3.92°), no significant difference was found among the three groups (F = 1.598, p > 0.001, η^2^ = 0.106). For the knee joint (FAI: 2.90° ± 0.52°, Coper: 3.30° ± 1.54°, CON: 2.89° ± 0.65°), no significant difference was observed (F = 0.513, p > 0.001, η^2^ = 0.037). For the ankle joint (FAI:22.89° ± 3.31°, Coper: 21.58° ± 4.13°, CON: 20.28° ± 2.54°), no significant difference was also found (F = 1.477, p > 0.001, η^2^ = 0.099).

The maximum joint angles are shown in Figure 3b. For the hip joint (FAI:31.21° ± 5.04°, Coper:34.20° ± 4.02°, CON:35.96° ± 3.58°), no significant difference was found (F = 3.178, p > 0.001, η^2^ = 0.191). For the hip joint (FAI:9.20° ± 1.55°, Coper:2.81° ± 1.46°, CON:2.07° ± 1.98°), a significant difference was found among the three groups (F = 54.581,p < 0.001, η^2^ = 0.801), with the FAI group significantly greater than the Coper and CON groups (p < 0.05). For the knee joint (FAI:1.17° ± 0.58°, Coper:0.87° ± 0.72°, CON:1.32° ± 0.52°), no significant difference was observed (F = 1.384, p > 0.001, η^2^ = 0.093). For the ankle joint (FAI: 1.38° ± 0.66°, Coper: 2.69° ± 0.85°, CON: 2.68° ± 0.89°), a significant difference was found (F = 8.756, p < 0.001, η^2^ = 0.393), with the FAI group significantly greater than the Coper and CON groups (p < 0.05).

For the ankle joint (FAI: 24.26° ± 3.52°, Coper: 24.27° ± 3.73°, CON: 22.96° ± 2.59°), no significant difference was also found (F = 0.518, p > 0.001, η^2^ = 0.037).

The minimum joint angles are shown in Figure 3c. For the hip joint (FAI: 9.20° ± 1.55°, Coper: 2.81° ± 1.46°, CON: 2.07° ± 1.98°), a significant difference was found among the three groups (F = 54.581,p < 0.001, η^2^ = 0.801), with the FAI group significantly smaller than the Coper and CON groups (p < 0.05). For the knee joint (FAI: −1.74° ± 0.35°, Coper: −2.43° ± 1.20°, CON: −1.57° ± 0.97°), no significant difference was observed (F = 2.430, p > 0.001, η^2^ = 0.153). For the ankle joint (FAI: 24.26° ± 3.52°, Coper: 24.27° ± 3.73°, CON: 22.96° ± 2.59°), no significant difference was also found (F = 0.518, p > 0.001, η^2^ = 0.037).

### Muscle synergies

#### Number of synergy modules

**FIGURE 4:**
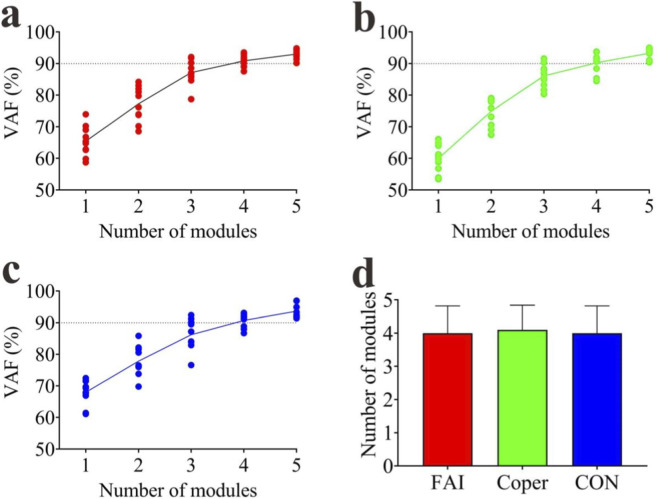
Number of synergy modules. **(a–c)** Represent the variance accounted for (VAF) corresponding to different numbers of synergy modules in the FAI, Coper, and CON groups, respectively. **(d)** Shows the comparison of the number of synergy modules among the three groups. FAI, Functional Ankle Instability group; Coper, Coper group; CON, healthy control group.

When the 90% VAF threshold was first reached, in the FAI group, 3 participants exhibited three modules, 4 exhibited four modules, and 3 exhibited five modules (Figure 4a). In the Coper group, 2 participants exhibited three modules, 5 exhibited four modules, and 3 exhibited five modules (Figure 4b). In the CON group, 3 participants exhibited three modules, 4 exhibited four modules, and 3 exhibited five modules (Figure 4c). No significant difference was found in the number of synergy modules among the three groups (FAI: 4.00 ± 0.82, Coper: 4.10 ± 0.74, CON: 4.00 ± 0.82; F = 0.053, p > 0.001, η^2^ = 0.004) (Figure 4d).

#### Activation coefficient (C)

**FIGURE 5:**
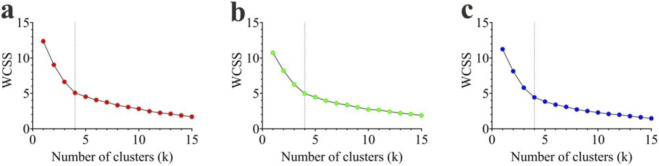
K-means clustering scree plots. **(a–c)** Represent the FAI, Coper, and CON groups, respectively.

**FIGURE 6:**
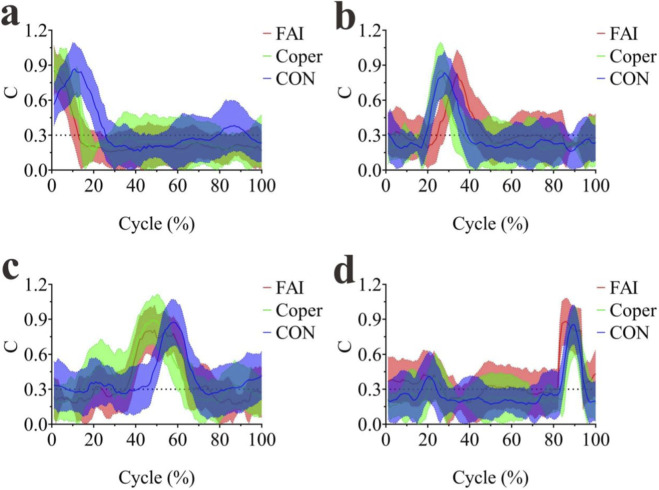
Activation curves of synergy modules. **(a–d)** Represent Modules 1–4, respectively. FAI, Functional Ankle Instability group; Coper, Coper group; CON, healthy control group; A one-way ANOVA was conducted to compare the activation duration of each module among the three groups. Significant differences were found in all four modules (p < 0.001) (Figure 7a). In Module1, the activation duration of the FAI and Coper groups was significantly shorter than that of the CON group, and the FAI group was significantly shorter than the Coper group (p < 0.05). In Module2, the FAI group was significantly longer than both the Coper and CON groups, and the Coper group was significantly shorter than the CON group (p < 0.05). In Module3, the FAI and Coper groups were significantly shorter than the CON group, and the FAI group was significantly shorter than the Coper group (p < 0.05). In Module4, the FAI group was significantly longer than both the Coper and CON groups (p < 0.05).

K- means clustering was performed on the synergy modules of all groups. The scree plot showed that the inflection point occurred at k = 4 for all three groups (Figure 5), indicating that the synergy modules could be classified into four clusters. The activation patterns of these four modules are shown in Figure 6. During the side-cutting movement following the jump-landing task, the muscles mainly served three functional roles in sequence: shock absorption during landing, propulsion during cutting, and maintenance of stability. Based on the shapes of the activation curves, Module1 peaked at the early phase of the movement and was primarily responsible for shock absorption; Module2 peaked in the early-to-mid phase, contributing to both shock absorption and propulsion; Module3 peaked in the mid phase, mainly related to propulsion; and Module4 peaked in the late phase, primarily responsible for stability maintenance.

**FIGURE 7:**
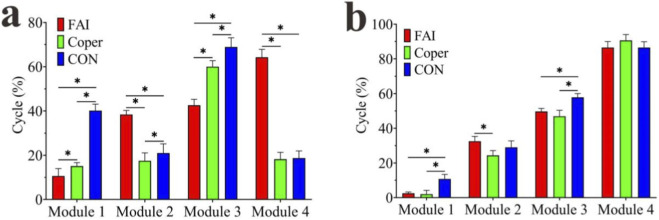
Comparison of activation curves. **(a)** Comparison of activation duration; **(b)** comparison of peak activation timing. FAI, Functional Ankle Instability group; Coper, Coper group; CON, healthy control group; * indicates that the p-value from multiple comparisons <0.05.

A one-way ANOVA was conducted to compare the peak activation timing of each module among the three groups. Significant differences were found in Modules1,2,and3 (p < 0.05) (Figure 7b). In Module1, the FAI and Coper groups reached peak activation significantly earlier than the CON group (p < 0.05). In Module2, the FAI group reached its peak significantly later than the Coper group (p < 0.05). In Module3, the FAI and Coper groups reached peak activation significantly later than the CON group (p < 0.05).

#### Main synergistic muscle (MSM)

**FIGURE 8:**
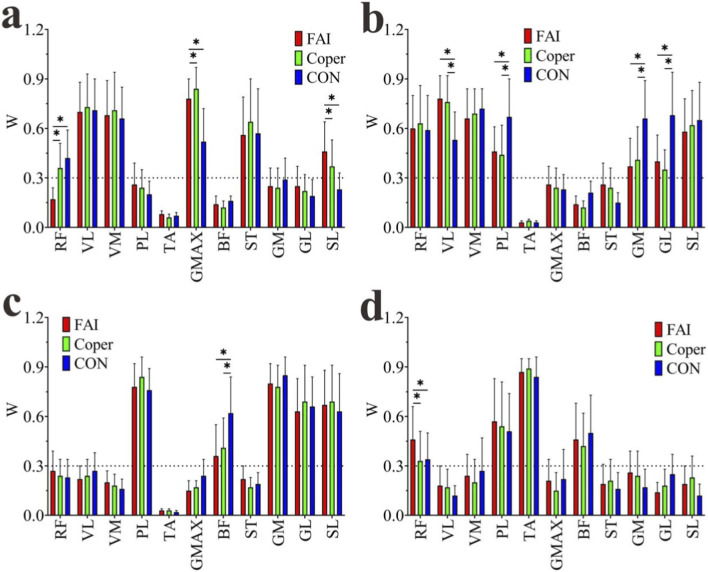
Muscle weightings of synergy modules. **(a–d)** Represent Modules 1–4, respectively. FAI, Functional Ankle Instability group; Coper, Coper group; CON, healthy control group; * indicates that the p-value from multiple comparisons <0.05.

The muscle weightings of each module are shown in Figure 8. Muscles with weightings greater than 0.3 were identified as the MSMs within each module. In Module1, the VL, VM, GMAX, and ST were shared MSMs across all groups; RF was a MSM in the Coper and CON groups; and SL was a MSM in the FAI and Coper groups. In Module2, the RF, VL, VM, PL, GM, ST, and SL were common MSMs in all groups. In Module3, the PL, BF, GM, ST, and SL were common MSMs across all groups. In Module4, the RF, PL, TA, and BF were common MSMs in all groups.

A one-way ANOVA was performed on the MSMs of each module, and significant differences were found in all four modules (p < 0.001). In Module1, the GMAX and SL of the FAI and Coper groups were significantly higher than those of the CON group, whereas RF was significantly lower than in the CON group (p < 0.05). In Module2, the VL of the FAI and Coper groups was significantly higher than that of the CON group, while the PL, GL, and GM were significantly lower than those in the CON group (p < 0.05). In Module3, the BF of the FAI and Coper groups was significantly lower than that of the CON group (p < 0.05). In Module4, the RF of the FAI group was significantly higher than those of both the Coper and CON groups (p < 0.05).

## Discussion

This study compared the lower-limb kinematic characteristics and muscle synergy patterns among individuals with functional ankle instability (FAI), copers (Coper), and healthy individuals (CON) during standing long jump-landing side-cut task, exploring the differences and compensatory mechanisms of neuromuscular control in FAI during dynamic movements. The results showed that individuals with FAI exhibited significantly increased knee and ankle joint range of motion in the sagittal plane, along with altered activation timing and weighting distribution of muscle synergies. These findings suggest that the neuromuscular control impairment exhibited by FAI is not a local problem but may reflect adaptive changes in neuromuscular control related to the CNS.

### Changes in kinematic characteristics and differences in control strategies

The results of this study indicated that, compared with the CON group and the Coper group, the FAI group exhibited significant differences in lower-limb kinematic characteristics during the standing long jump-landing side-cut task, with remarkable variations particularly observed in the flexion-extension control of the ankle and knee joints. Specifically, the FAI group showed notably larger ankle flexion-extension angles and a greater range of knee motion in the sagittal plane, suggesting that they adopt a “delayed cushioning-overflexion” movement strategy. This kinematic feature is considered an active protective adaptation developed by individuals with FAI under the condition of limited proprioceptive input, which works by prolonging the support phase, dispersing impact loads, and reducing ankle inversion torque to enhance stability (Gutierrez et al., 2009).

The moment of landing is a key phase for energy absorption and neuromuscular reflex control of the lower-limb joints (Yeow et al., 2011). In healthy individuals, energy absorption is achieved sequentially through coordinated ankle–knee motion, maintaining both stability and turning efficiency. However, this study found that the FAI group exhibited a greater knee flexion angle during this phase, indicating reduced ankle energy absorption capacity and an upward shift of the energy transfer center along the kinetic chain toward more proximal joints. This “knee-dominant absorption mode” reflects a redistribution of the neuromuscular system in response to ankle instability, a “proximal compensation” strategy (Kim et al., 2019). This strategy can reduce ankle load and pain in the short term but may alter lower-limb load distribution in the long term, decreasing energy efficiency and leading to functional fatigue in the knee and hip joints (Xu et al., 2022). Regarding the increased range of hip motion, it indicates that individuals with FAI stabilize their posture by enhancing the activity of proximal joints throughout the landing process.

In the frontal plane, this study found that the FAI group exhibited a significantly greater maximum hip adduction angle and a smaller ankle eversion angle compared with the other two groups. The increased hip adduction angle suggests that individuals with FAI tend to narrow their support base medially during landing, which may be a subconscious attempt to reduce the lateral lever arm of the body’s center of mass relative to the ankle joint—an instinctive response to avoid excessive ankle inversion that could trigger instability. Meanwhile, the reduced ankle eversion angle directly reflects the impaired active eversion capacity of the FAI group, as the ankle musculature fails to generate sufficient eversion torque to counteract the inherent inversion tendency during landing.

A smaller ankle eversion angle limits the ankle’s ability to adapt to ground irregularities and absorb lateral impact loads, thereby increasing the reliance on proximal joints (such as the hip) for balance regulation (Hof et al., 2005). This kinematic combination—greater hip adduction coupled with reduced ankle eversion—indicates that FAI individuals transfer the burden of frontal plane stability from the impaired ankle to the hip joint. However, prolonged reliance on increased hip adduction often places excessive compressive stress on the medial compartment of the knee joint and stretches the lateral hip abductors, while the persistently reduced ankle eversion may further weaken the peroneal muscle group through disuse, forming a vicious cycle of functional impairment (Delahunt et al., 2006). This finding further indicates that although the compensatory strategy of FAI (shifting stability control to proximal joints) may temporarily prevent acute ankle sprains, it does so at the expense of normal joint load distribution and long-term musculoskeletal health.

It is worth noting that compared with healthy individuals, the FAI group’s enhanced hip functional involvement (manifested as increased adduction) and reduced ankle eversion collectively reflect the reorganization of their frontal plane control pattern. Normally, the ankle first perceives lateral impact and activates the peroneal muscles to generate eversion torque, achieving preliminary frontal plane stabilization. However, in individuals with FAI, the weakened peroneal muscle response leads to insufficient ankle eversion, and the delayed sensory feedback prompts the central nervous system to recruit the hip adductors and medial rotators earlier in the control chain. This “proximal-prioritized activation sequence” replaces the normal “distal-to-proximal” control flow, which is considered a key marker of neural control adaptation in FAI (Kim et al., 2019).

Thus, these kinematic characteristics—including the increased range of motion of the knee and ankle joints in the sagittal plane, as well as the increased hip adduction and decreased ankle eversion in the coronal plane—are not merely external manifestations at the joint displacement level, but more importantly, reflect the in-depth reorganization of neural drive patterns related to motor control. The transition of lower limb stability control from an “ankle-dominant” mode to a “proximal knee-hip dominant” mode not only highlights the adaptive nature of the neuromuscular system in response to ankle functional impairments but also reveals the new biomechanical risks associated with this compensatory process, such as joint load imbalance and long-term injuries.

### Muscle synergy patterns, neuromuscular control characteristics and related mechanisms

The muscle synergy analysis in this study showed that when reaching 90% VAF, the number of synergy modules was the same among the FAI, Coper, and CON groups, indicating that the dimensionality of overall neural control required to complete the moderate-demand standing long jump-landing side-cut task remained consistent. This result suggests that FAI does not cause structural degeneration of the control system—there is no reduction in the number of control modules. However, the FAI group exhibited significant alterations in the temporal and sequential distribution of synergy activations, showing evident time rearrangement. This temporal disorder precisely reflects the neural driving abnormality related to movement execution.

Specifically, individuals with FAI showed significantly shortened synergy activation durations during the landing cushioning phase (Module 1) and mid-force phase (Module 3), while showing prolonged durations during the transition (Module 2) and terminal stabilization (Module 4) phases—forming a typical “insufficient early activation and delayed compensation” pattern. This change in synergy timing indicates that individuals with FAI lack rapid response capability during the initial landing phase, leading to insufficient early dynamic precision. Correspondingly, a “delayed defense” emerged to compensate for the initial insufficiency, prolonging muscle contractions in the later phases to stabilize the body’s center of mass (Kim et al., 2023). This delayed control pattern causes an overall posterior shift in movement timing, reducing neuromuscular response efficiency (Delahunt et al., 2006).

Notably, the EMG weight distribution across modules presents significant specific differences: in Module 1, the rectus femoris (RF) weight of the FAI group was significantly lower, while the weights of the gluteus maximus (GMAX) and soleus (SL) were significantly higher; in Module 2, the vastus lateralis (VL) weight was significantly increased, whereas the weights of the peroneus longus (PL), gastrocnemius (GM), and gluteus medius (GL) were significantly decreased; in Module 3, the biceps femoris (BF) weight was significantly lower; and in Module 4, the rectus femoris (RF) weight was significantly higher.

These module-specific changes in EMG weights further refine the overall trend of “proximal dominance and distal insufficiency”. From a functional perspective, the high weights of GMAX and SL in Module 1 (landing cushioning) reflect the early involvement of proximal hip muscles and deep calf stabilizers to enhance cushioning stability; the high activation of VL and low contribution of PL and GM in Module 2 (transition phase) indicate a regulatory mechanism where knee muscles compensate for the distal control deficits of the ankle joint; the reduced BF weight in Module 3 (mid-force phase) impairs the dynamic regulatory function of knee flexors, while the increased RF weight in Module 4 (terminal stabilization) strengthens the final postural stability by enhancing the activity of knee extensors. Distal muscles are mainly responsible for rapid reflexes and fine adjustments, while proximal muscles (such as hip extensors and deep stabilizers) primarily function in postural maintenance and energy absorption (Rios et al., 2015). When individuals with FAI cannot rely on ankle muscles for adequate dynamic control during landing, the nervous system recruits proximal drives through such module-specific muscle weight restructuring and adjusts the activation levels of key knee muscles to increase limb stiffness and maintain stability. Therefore, this control shift serves as both a protective mechanism and an adaptive adjustment to impaired ankle sensory input.

This neuromuscular adjustment has dual functional implications. On one hand, increased proximal activation enhances overall postural control and limb stiffness, allowing FAI individuals to effectively prevent recurrent sprains or instability during movement—a “safety-first” strategy (Rios et al., 2015). On the other hand, weakened distal rapid response inevitably delays the early response phase, reduces control precision, and impairs motion fluidity and efficiency (Delahunt et al., 2006). Specifically, in high-frequency or sudden directional-change tasks, this control mode appears as slowed reactions, rigid posture, and reduced shock absorption capacity (Simpson et al., 2019). Hence, FAI individuals ensure stability at the cost of speed and efficiency—an energy-expensive stabilization strategy.

Further analysis revealed a up-bottom substitution trend in neuromuscular drive among FAI individuals. Due to impaired proprioceptive transmission at the ankle, distal feedback becomes delayed, causing temporal misalignment of control signals. The CNS compensates by preactivating proximal muscles (such as the gluteus maximus and biceps femoris), shifting the energy transfer chain from a “distal-driven” to a “proximal-initiated” model. This aligns with neural plasticity regulation principles—when local input is impaired, the system reconstructs functional control networks by adjusting signal pathways and muscle couplings (Vélez-Gutiérrez et al., 2022; Kwon et al., 2020; Kweon et al., 2022). Although the total number of synergy modules remains constant, their temporal sequencing and weight distribution are reconfigured, forming an asymmetric control structure.

From a biomechanical control perspective, the temporal reconstruction in FAI reflects phase dysregulation of neuromuscular drive signals (Pelletier et al., 2015). Normal motion control relies on precise inter-muscle timing to achieve optimal energy transfer. However, due to impaired ankle proprioceptive transmission, the timing of neuromuscular signals becomes misaligned, disrupting synergy synchronization. As a result, movement rhythm and agility decline, while compensatory rigidity increases. Although this ensures stability, it weakens adaptability and may lead to stereotyped response patterns over time.

In summary, the abnormal synergy control in FAI can be summarized in three aspects: (1) synergy quantity remains stable, but internal timing is reorganized; (2) control drive shifts from distal to proximal dominance, reflecting compensatory stabilization; and (3) reduced efficiency and asynchronous timing are associated with altered movement control. These findings reveal that the neuromuscular control deficit in FAI is not structural degeneration but a spatiotemporal adaptive adjustment process.

The abnormal neuromuscular control in functional ankle instability is closely related to impaired peripheral proprioceptive input and adaptive adjustments of neuromuscular control (Boudreau et al., 2010). Various mechanoreceptors in ligaments and joint capsules are damaged after sprain, delaying position-sense transmission and weakening spinal reflex modulation and rapid correction ability (Konradsen and Ravn, 1991). When peripheral feedback is insufficient, the CNS must reorganize its control strategies by enhancing proximal activation and feedforward outputs to compensate for feedback delays (Pelletier et al., 2015). The delayed early activation observed in the FAI group corresponds to this biomechanical adaptation.

However, this compensatory strategy has side effects—greater energy consumption and earlier muscle fatigue (Kim et al., 2019). The landing task requires rapid energy release and absorption, but FAI individuals over-recruit large muscles during high-intensity efforts, causing stiffness and excessive co-activation. Research indicates that such stiffness reduces motion flexibility, delays relaxation, and produces a “motion inertia” phenomenon (Ford et al., 2008). The prolonged activation in the late phase observed here reflects this mechanism, suggesting insufficient “muscle shut-off” response, where the nervous system remains hyperactivated post-task, delaying relaxation. Overall, the neuromuscular control pattern of FAI individuals forms a “delay-compensated” feature.

To more comprehensively interpret the observed kinematic and muscle synergy changes in individuals with FAI, it is necessary to acknowledge the limitation of this study–the lack of joint coordination pattern analysis, which is an important dimension in contemporary landing biomechanics research. Joint coordination (e.g., hip-knee, knee-ankle planar coupling) directly reflects inter-joint control strategies, and methods such as vector coding or continuous relative phase have become standard approaches for interpreting motor coordination in FAI and Coper populations (Kwon et al., 2020; Kweon et al., 2022). For example, altered ankle-shank coupling during running was found in individuals with Chronic Ankle Instability (CAI), and differences in foot-shank coordination between Copers and CAI individuals during walking were confirmed. These studies highlight that inter-joint coordination abnormalities are core features of ankle instability, which may underlie the “proximal compensation” observed in the FAI group in this study.

Due to the absence of such coordination metrics in the current analysis, interpretations of “system-level reorganization” should be cautious. The observed kinematic and muscle synergy changes are more likely to reflect local compensatory adjustments rather than comprehensive systemic reorganization. Future studies will integrate joint coordination analysis to more fully capture the overall motor control characteristics of FAI.

## Conclusion

During standing long jump-landing and side-cutting tasks, individuals with FAI exhibit distinct kinematic and neuromuscular control characteristics. Kinematically, FAI participants demonstrate a protective movement adjustment characterized by increased range of motion of the ankle and knee joints in the sagittal plane, accompanied by greater hip adduction and reduced ankle eversion in the frontal plane Muscle synergy analysis reveals that, although the number of synergy modules is comparable to that of the control group, FAI individuals display an asymmetric temporal pattern typified by insufficient early activation and prolonged late-phase compensation, as well as a proximal-dominant and delayed distal-response control mode. In contrast, Copers show kinematic and synergy patterns similar to those of healthy individuals, yet still exhibit slight over-flexion and delayed timing, suggesting that their distal feedback function has partially recovered, though residual protective responses remain evident. These findings hold significant significance: they suggest that FAI rehabilitation should focus on strengthening distal ankle muscles and enhancing proximal–distal coordination to improve joint control, while also emphasizing the need for more sensitive dynamic assessment methods to identify potential instability risks.

## Data Availability

The raw data supporting the conclusions of this article will be made available by the authors, without undue reservation.
